# A sex- and gender-based analysis of factors associated with linear growth in infants in Ecuadorian Andes

**DOI:** 10.1038/s41598-022-06806-3

**Published:** 2022-02-28

**Authors:** Emily C. Evers, William F. Waters, Carlos Andres Gallegos-Riofrío, Chessa K. Lutter, Christine P. Stewart, Lora L. Iannotti

**Affiliations:** 1 Brown School, Washington University in St. Louis, St. Louis, MO USA; 2 Instituto de Investigación en Salud Y Nutrición, Universidad San Francisco de Quito, Quito, Ecuador; 3 Gund Institute for the Environment, Rubenstein School of Environment and Natural Resources, University of Vermont, Burlington, VT USA; 4 RTI International, Washington, DC USA; 5 Department of Nutrition, University of California Davis, Davis, CA USA; 6 Institute for Public Health, Washington University in St. Louis, St. Louis, MO USA

**Keywords:** Biomarkers, Risk factors, Signs and symptoms, Diseases, Nutrition disorders, Malnutrition

## Abstract

Although female infants may have an early life biological advantage over males, gendered treatment can alter health outcomes. Ecuador has an unusually high ratio of male to female infant mortality, but gender norms have been reported to favor boys. This analysis of baseline data from the Lulun Project, a randomized controlled trial conducted in rural Andean communities of Ecuador, investigates the roles of sex and gender in undernutrition among infants 6 to 9 months of age. Twenty-four-hour recall frequencies were used to assess dietary intake. Food outcome models were analyzed as prevalence ratios calculated using a binomial distribution with a log link or robust Poisson regression. Linear regression was used to analyze the continuous growth outcome length-for-age *z* score. Socioeconomic and health history variables were comparable between male and female infants. Boys were more often fed liquids other than breastmilk within their first 3 days of life (17.1% vs. 5.2%, *P* = 0.026). Compared with girls, boys were less likely to be fed eggs by 33% (95% CI 0.46, 0.96), cheese, yogurt, or other milk products by 40% (95% CI 0.39, 0.92), yellow fruit by 44% (95% CI 0.33, 0.97), water by 37% (95% CI 0.45, 0.88), thin porridge by 29% (95% CI 0.56, 0.92), and tea without milk by 67% (95% CI 0.11, 0.99). Prevalence of boys with an adequate dietary diversity score (≥ 4) was reduced by 27% relative to girls (95% CI 0.54, 0.99). Males fared worse in length-for-age *z* scores (− 2.16 vs. − 1.56, *P* = 0.000), weight-for-age *z* scores (− 0.86 vs. − 0.33, *P* = 0.002), prevalence of stunting (50.6% vs. 23.4%, *P* = 0.000), and plasma concentrations of dimethylglycine (1.25 vs. 1.65 µg/mL, *P* = 0.021). After adjusting for demographic, caregiver perceptions of appetite, and biological factors, length-for-age *z* score for a male child was 0.62 units lower than for a female (95% CI − 0.98, − 0.26). Male infants were shown to receive lower quality complementary foods and have worse anthropometric measures than female infants.

*Trial registration* clinicaltrials.gov, NCT02446873. Registered February 28, 2015, https://clinicaltrials.gov/ct2/show/NCT02446873.

## Introduction

From infancy through early childhood, across a variety of contexts and health outcomes, females have been observed to have a biological advantage over males^1–5^. In 2012, the global infant mortality sex ratio (the ratio of male to female mortality) was 1.13 (90% uncertainty interval, 1.12–1.15), indicating 13% more infant deaths among males than females worldwide^6^. A recent systematic review and meta-analysis finds that this pattern extends to undernutrition; boys under age 5 are more likely to experience wasting, stunting, and underweight than girls^7^. However, these results are context dependent. Girls’ early life advantage is predicated upon equal treatment. In contexts where boys are valued over girls and given greater access to health care and nutrition, the biological female advantage may be diminished or even reversed^8^.

Although in many contexts, males are expected to have higher rates of morbidity and mortality, extremely high mortality sex ratios may indicate excess male deaths (i.e., more deaths than would be expected by biological factors alone). Ecuador, with an unusually high ratio of male to female infant mortality of 1.32, may have a greater-than-expected degree of male disadvantage in survival^5^, which may imply a gendered behavioral effect. This difference would be significant because gender inequality that favors men has been identified as a key factor impacting Ecuador’s standing on the Human Development Index^9^. Moreover, in traditional gender-based practices in highland indigenous communities in Ecuador, lactation and weaning practices favored boys^10^. Given these opposing trends, it is unclear whether, in this context, one would expect differences in breastfeeding and complementary feeding, which contribute to infant survival, to favor boys or girls.

From March to December 2015, the Lulun Project, a randomized controlled trial (RCT), was conducted in rural Andean communities in Ecuador to examine the effects of egg consumption early in the complementary feeding period on nutritional outcomes^11^. At baseline, we observed greater prevalence of undernutrition among male infants. To better understand this observation, the objective of the present study was to investigate the roles of both sex (biological attributes) and gender (social constructs)^12^ in undernutrition. The latter refers to not only how individuals identify themselves but also how boys and girls (including infants) are treated by others. We tested whether differences in breastfeeding and complementary feeding practices exist, as well as their relation to biological markers and growth outcomes. Given reports of gender inequality in Ecuador as well as its outlying infant mortality sex ratio, we hypothesized that gendered differences in breastfeeding and complementary feeding were likely to exist.

Throughout this report, we attempt to thoughtfully use the terms sex and/or gender in accord with the Sex and Gender Equity in Research (SAGER) guidelines^12^. In practice, even with careful consideration, these terms may prove difficult if not impossible to disentangle. Often, outcomes may be a mixture of both biological effects of sex combined with social and behavioral impacts of gender^13^. In this report, when focusing on biological factors (e.g., report of biomarkers), we use the term sex. When discussing factors that imply a behavioral or social component (e.g., complementary feeding behaviors or practices), we use the term gender. In doing so, we do not intend to convey a strict separation; indeed, we acknowledge that in many cases they are inseparable. When considering both biological along with social and behavioral factors, we use the combined term, sex/gender (however, we do not intend for this to suggest that the terms are interchangeable). We acknowledge, as have previous authors, definitions of sex and gender are evolving as science in this area grows^13^.

## Methods

### Study sample

This study employs baseline data collected for the Lulun Project, an RCT that investigated nutritional outcomes in children after an intervention that promoted greater consumption of eggs^11^. The study took place in 5 rural parishes of Cotopaxi Province, Ecuador. This region has a large indigenous population; according to National Census Data, approximately one-fifth (22%) of Cotopaxi’s population of 409,205 persons self-identified as indigenous compared to 7% nationally^14^. Approval for the study was obtained from ethical oversight committees at the Universidad San Francisco de Quito, Washington University in St. Louis, and the Pan American Health Organization. All mothers or primary caregivers provided written informed consent prior to data collection. All research was performed in accordance with the Declaration of Helsinki.

Study design and recruitment methods have been described in detail previously^11,15^. Eligibility requirements for infants included good health, born singleton, and aged 6 to 9 months at the time of recruitment. Exclusion criteria included congenital heart condition, severe acute malnutrition, and egg allergy. Potential eligible mother or other caregiver-infant pairs were transported to data collection sites. Of the initial 179 pairs assessed for eligibility, 4 did not meet eligibility criteria. An additional 9 caregivers declined for reasons such as moving from the area, concerns regarding blood draws, or other reasons. This analysis is based on 166 mother or other caregiver-infant pairs who enrolled in the study and completed baseline questionnaires. Biomarker data is available for 142 infants.

### Study variables

Mothers or other caregivers provided socioeconomic, demographic, breastfeeding status, child dietary intake, and morbidity information via an administered survey. Foods commonly consumed in the area were determined through formative research^16,17^. Twenty-four-hour recall frequencies were used to assess dietary intake and to assign a dietary diversity score^18^. Morbidities were assessed through 7-day recall, focusing on conditions prevalent in the area, including diarrheal and respiratory symptoms, as well as fever, skin rash, persistent cough, congestion or runny nose, panting, wheezing, or difficulty breathing, and toothache or teething.

#### Biomarkers

Plasma concentrations of nutrient biomarkers, including choline, betaine, dimethylglycine (DMG), vitamin B12, linoleic acid (LA; 18:2n-6), α-linolenic acid (ALA; 18:3n-3), and docosahexaenoic acid (DHA) were analyzed. Specimen processing and laboratory methods have been described previously^19^. In brief, nutrient concentrations for choline, betaine, DMG, LA, ALA, and DHA were determined by modified liquid chromatography-tandem mass spectrometry (LC–MS/MS) methods. For vitamin B12 concentrations, chemiluminescent competitive immunoassay was used (IMMULITE 1000 Analyzer; Diagnostic Products).

#### Growth outcomes

International standardized protocols were used to guide measurement of anthropometric data^20^. As previously described by Iannotti et al. (2017), study enumerators participated in 3-day training and validation exercises. Enumerator pairs each took measurements of child length to the nearest 1 mm using a Seca 417 portable infantometer. If measures differed by 5 mm or more, a third measurement was made, and the result was averaged with the closest measure. A Seca Model 874 Electronic Digital Scale, with mother/child tare feature to the nearest 0.01, was used to measure weight. If the two measures taken differed by 0.05 kg or more, a third measure was made, and the results averaged. Measures were converted to *z* scores for length-for-age (LAZ), weight-for-age (WAZ), weight-for-length (WLZ), and BMI (BMIz)^11^. Stunting is defined as length-for-age *z* score (LAZ) < − 2^20^.

### Statistical methods

Tables were generated to estimate associations between the exposure groups of interest (girls and boys) and possible confounders and then to compare responses to food frequency questionnaires across the groups. For bivariate statistics, probability values were obtained with a Fisher's exact test for categorical variables and two-sample *t* test for continuous variables. When unequal variances were detected, a two-sample *t* test with unequal variances was used. For continuous variables, when deviations from normality were detected, results were rechecked with a two-sample Wilcoxon rank-sum test.

Food outcome models (dichotomous outcomes) were analyzed as prevalence ratios calculated using a binomial distribution with a log link or robust Poisson regression when a model failed to converge^21^. We first fit an unadjusted model including only child’s gender, then adjusted for child’s age, mother’s age and education. We used the Benjamini–Hochberg method with a false discovery rate threshold of 0.20 to adjust for multiple comparisons across individual food items^22^.

Linear regression was used to analyze our continuous growth outcome LAZ. We first fit an unadjusted model including only child’s sex/gender. In our adjusted model, we included demographic variables (child’s sex/gender, age, and firstborn status, and maternal age and education), breastfeeding and complementary feeding variables (fed something other than breast milk within the first three days, child’s appetite, and dietary diversity score ≥ 4), and biomarkers (choline, betaine, DMG, vitamin B12, ALA, and DHA). We did not include the biomarker LA because of a high correlation (*r* = 0.878) with ALA. We tested for a sex/gender and vitamin B12 interaction, added to the adjusted model. We conducted diagnostic and residual analysis on the adjusted model. Problematic multicollinearity was not detected based on variance inflation factors (VIF); VIFs were < 2 for all variables. A Breusch-Pagan/Cook-Weisberg test for heteroskedasticity was nonsignificant (*P* = 0.179). Residuals were assessed graphically and appear reasonably normally distributed. Influential cases were examined using Cook’s distance; no observations were identified as influential. All analyses were performed using STATA software (version 14.2; StataCorp), and *P*-values ≤ 0.050 were considered statistically significant.

### Ethics approval and consent to participate

Approval for the study was obtained from ethical oversight committees at the Universidad San Francisco de Quito, Washington University in St. Louis, and the Pan American Health Organization. All mothers or primary caregivers provided written informed consent prior to data collection.

## Results

Of the 166 children included in the study, 54% were male. No statistically significant differences were observed between groups in terms of child age or percent firstborn (Table 1). The groups were similar regarding maternal characteristics, including maternal age, teenage mother, years of education completed, maternal employment outside the home, and type of caregiver reporting. No statistically significant differences were observed regarding household or socioeconomic factors, including number of household members, types of crops grown, livestock owned, participation in the government’s conditional cash transfer program, use of an improved water source, treated water, flush toilet, and gas or electric cooking fuel.

**Table 1:** Characteristics of study children by gender.

Characteristics	Girls (*n* = 77)	Boys (*n* = 89)	*P* value^a^
**Child**			
Age, mo	7.72 ± 1.2	7.45 ± 1.1	0.130
Firstborn, %	27 (35.1%)	41 (46.1%)	0.159
**Maternal**			
Maternal age, y	26.2 ± 6.9	24.8 ± 6.5	0.171
Teenage mother (19 y or less), %	14 (18%)	23 (26%)	0.266
Education completed, y	8.9 ± 2.9	9.1 ± 3.2	0.707
Mother employed outside home, %	37 (48%)	38 (43%)	0.533
**Caregiver reporting, %**			
Mothers	67 (87%)	74 (83%)	0.450
Grandmothers	6 (8%)	6 (7%)	
Aunts	3 (4%)	4 (4%)	
Fathers	0 (0%)	4 (4%)	
Sisters	1 (1%)	1 (1%)	
**Household**			
No. of household members	6.0 ± 2.2	6.2 ± 2.2	0.537
**Food production, %**			
Any crops	63 (82%)	76 (85%)	0.674
Tubers	59 (77%)	67 (75%)	0.858
Legumes	38 (49%)	39 (44%)	0.534
Fruit or vegetables	37 (48%)	49 (55%)	0.437
**Livestock ownership, %**			
Any livestock	66 (86%)	74 (83%)	0.675
Poultry	46 (60%)	51 (57%)	0.755
Cows	28 (36%)	37 (42%)	0.526
Sheep, goats	17 (22%)	18 (20%)	0.849
Guinea pigs, rabbits	52 (68%)	51 (57%)	0.201
**Conditional cash transfer program (Bono de Desarrollo Humano), %**^b^	20 (27%)	22 (25%)	0.858
**Improved water source, %**	75 (97%)	89 (100%)	0.214
**Treated water, %**	61 (79%)	61 (69%)	0.158
**Flush toilet, %**	68 (88%)	79 (89%)	1.000
**Gas or electricity cooking fuel, %**^c^	63 (86%)	74 (84%)	0.825

Data are presented as mean ± standard deviation (SD) and Number (%).

^a^Fisher's exact test for categorical variables and two-sample *t* test for continuous variables.

^b^*n* = 163.

^c^*n* = 161.

We compared the groups for differences in health characteristics (Supplemental Table 1). No statistically significant differences were observed in 6-month histories of health service visits (to public hospitals or health centers, private clinics, or others). Similarly, no statistically significant differences were found in 6-month use of supplements or other nutrition products (iron, vitamin A, multivitamins or minerals, or food rations). The two groups were similar in report of 7-day histories of fever, diarrhea, blood in stool, skin rash, constant cough, congestion/runny nose, panting/wheezing/difficulty breathing, bruising, scrapes, or cuts, toothache/teething, and receipt of oral rehydration salts.

A similar proportion of infant boys and girls were ever breastfed, breastfed within 1 h of birth, fed colostrum, received practical support or advice to start breastfeeding, breastfed yesterday, and who the decision-maker was for what the child should and should not be fed (Table 2). But boys were reported 3 times more often than girls to have been fed something other than breast milk within their first 3 days of life. Differences were also noted in caregiver’s assessment of the child’s appetite, with poor appetite reported nearly 5 times less frequently for boys than girls.

**Table 2:** Infant feeding by gender.

	Girls (*n* = 77)	Boys (*n* = 89)	*P* value^a^
**Ever breastfed**	77 (100%)	88 (98.9%)	1.000
**Breastfed within 1 Hour of Birth**^b^	42 (54.6%)	46 (52.3%)	0.876
**Fed colostrum**^c^	72 (97.3%)	85 (96.6%)	1.000
**First 3 days fed something other than breastmilk**^b^	4 (5.2%)	15 (17.1%)	0.026
Food given:			
Tea or herbal infusion	0 (0%)	1 (6.7%)	1.000
Water (includes sugar water)	1 (25.0%)	2 (13.33%)	
Infant formula	3 (75.0%)	12 (80.0%)	
**During first 3 days, received practical support or advice to help start breastfeeding**^d^	37 (48.7%)	53 (60.9%)	0.155
**Breastfed yesterday**	75 (97.4%)	87 (97.8%)	1.000
**Who mainly decides what the child should and should not eat?**			
The mother	72 (93.5%)	83 (93.3%)	1.000
A grandparent	4 (5.2%)	5 (5.6%)	
The father	1 (1.3%)	1 (1.12%)	
**Generally speaking, how is child's appetite when healthy?**			
Eats too much	13 (16.9%)	15 (16.9%)	0.024
Eats well	52 (67.5%)	71 (79.8%)	
Eats a little	12 (15.6%)	3 (3.4%)	

Data are presented as Number (%).

^a^Fisher's exact test for categorical variables.

^b^*n* = 165.

^c^*n* = 162.

^d^*n* = 163.

The prevalence ratios of boys receiving the same food items within the last 24 h as compared to girls are reported in Table 3. In all cases, when a statistically significant difference was detected, it favored receipt of the item by girls. In adjusted models, prevalence of receiving the following food items were reduced for boys relative to girls: by 33% for eggs, 40% for cheese, yogurt, or other milk products, 37% for water, 29% for thin porridge, 67% for tea without milk, and 44% for yellow fruit. Prevalence of boys receiving a dietary diversity score of ≥ 4 was reduced by 27% relative to girls. These results remained significant after applying the Benjamini–Hochberg procedure with a 0.20 false discovery rate level. No statistically significant differences were detected among other food items.

**Table 3:** Food item consumption of study children by gender.

	Girls (*n* = 77)	Boys (*n* = 89)	Prevalence ratios (PR) and 95% confidence intervals (CIs) for boys compared to girls (reference)					
			Unadjusted^a^			Adjusted^a,b^		
Consumed Last 24 h	N (%)	N (%)	PR	95% CIs	*P* value	PR	95% CIs	*P* value
**Animal source foods**								
Eggs	38 (49.4%)	30 (33.7%)	0.68	0.47, 0.99	0.043^d^	0.67	0.46, 0.96	0.030^d^
Milk	9 (11.7%)	17 (19.1%)	1.63	0.77, 3.45	0.198	1.65	0.77, 3.53	0.195
Other dairy (cheese, yogurt, or other milk products)	31 (40.3%)	22 (24.7%)	0.61	0.39, 0.97	0.035^d^	0.60	0.39, 0.92	0.019^d^
Fish (fresh or dried fish, shellfish, or seafood)	14 (18.2%)	14 (15.7%)	0.87	0.44, 1.70	0.674	0.84	0.42, 1.65	0.607
Organ meat (liver, kidney, heart, or other organ meat)	17 (22.1%)	14 (15.7%)	0.71	0.38, 1.35	0.298	0.76	0.40, 1.44	0.399
Any meat (beef, pork, lamb, goat, chicken, or duck)	46 (59.7%)	42 (47.2%)	0.79	0.59, 1.05	0.106	0.83	0.64, 1.09	0.183
**Legumes (any foods made from beans, peas, lentils, nuts, or seeds)**	7 (9.1%)	13 (14.6%)	1.61	0.68, 3.82	0.284	1.79	0.79, 4.05	0.165
**Oils, fats, or butter (or foods made from any of these)**	18 (23.4%)	24 (27.0%)	1.15	0.68, 1.96	0.597	1.24	0.73, 2.10	0.421
**Grains (porridge, bread, rice, noodles, or other foods made from grains)**	53 (68.8%)	56 (62.9%)	0.91	0.73, 1.14	0.422	0.94	0.75, 1.16	0.553
**Liquids**								
Water	46 (59.7%)	31 (34.8%)	0.58	0.42, 0.82	0.002^d^	0.63	0.45, 0.88	0.007^d^
Infant formula	12 (15.6%)	20 (22.5%)	1.44	0.75, 2.75	0.268	1.51	0.79, 2.88	0.208
Fruit juice^c^	30 (39.0%)	31 (35.2%)	0.90	0.61, 1.35	0.620	0.96	0.66, 1.39	0.832
Clear broth	70 (90.9%)	77 (86.5%)	0.95	0.85, 1.06	0.371	0.96	0.87, 1.07	0.494
Thin porridge	55 (71.4%)	44 (49.4%)	0.69	0.54, 0.89	0.004^d^	0.71	0.56, 0.92	0.009^d^
Tea with milk	2 (2.6%)	7 (7.9%)	3.03	0.65, 14.15	0.159	3.01	0.64, 14.22	0.164
Tea without milk	11 (14.3%)	4 (4.5%)	0.31	0.10, 0.95	0.040^d^	0.33	0.11, 0.99	0.048^d^
Coffee	9 (11.7%)	7 (7.9%)	0.67	0.26, 1.72	0.409	0.69	0.27, 1.76	0.434
Soda	5 (6.5%)	4 (4.5%)	0.69	0.19, 2.49	0.573	0.83	0.23, 2.97	0.781
Other sugary drinks	4 (5.2%)	9 (10.1%)	1.95	0.62, 6.07	0.251	1.94	0.62, 6.07	0.254
Other liquids^c^	26 (33.8%)	22 (25.0%)	0.74	0.46, 1.19	0.218	0.78	0.48, 1.27	0.32
**Fruits and vegetables**								
Dark green leafy vegetables	13 (16.9%)	13 (14.6%)	0.87	0.43, 1.75	0.687	0.90	0.45, 1.79	0.772
Yellow fruit (ripe mangoes, ripe papayas, or tomato from the tree or any other yellow meat fruit)^c^	25 (32.5%)	16 (18.2%)	0.56	0.32, 0.97	0.038^d^	0.56	0.33, 0.97	0.040^d^
Root vegetables (white potatoes, white yams, manioc, cassava, or other foods made from roots)	35 (45.5%)	44 (49.4%)	1.09	0.79, 1.50	0.610	1.06	0.78, 1.46	0.696
Yellow vegetables (pumpkin, carrot, squash, or sweet potatoes that are yellow or orange inside)	22 (28.6%)	22 (24.7%)	0.87	0.52, 1.44	0.575	0.98	0.61, 1.58	0.95
Other Fruits or vegetables	41 (53.3%)	47 (52.8%)	0.99	0.74, 1.32	0.955	1.00	0.75, 1.34	0.985
**Sugary foods (any sugary foods, such as chocolates, sweets, candies, pastries, cakes, or biscuits)**	17 (22.1%)	30 (33.7%)	1.53	0.92, 2.54	0.104	1.46	0.87, 2.46	0.149
**Condiments (for flavor, such as chilies, spices, herbs, or fish powder)**	27 (35.1%)	28 (31.5%)	0.90	0.58, 1.38	0.622	0.93	0.59, 1.46	0.746
**Grubs, snails, or insects**	0 (0%)	3 (3.4%)						
**Red Palm (foods made from red palm oil, red palm nut, or red palm nut pulp sauce)**	15 (19.5%)	16 (18.0%)	0.92	0.49, 1.74	0.804	0.93	0.50, 1.72	0.81
**Dietary Diversity Score ≥ 4**	45 (58.4%)	36 (40.5%)	0.69	0.51, 0.95	0.022^d^	0.73	0.54, 0.99	0.045^d^

^a^Calculated with log binomial or robust Poisson regression.

^b^Adjusted for child's age, mother's age, and mother's education.

^c^*n* = 165.

^d^Remained significant at 0.2 false discovery rate.

With regard to anthropometric measures LAZ and WAZ, males fared significantly worse than females; LAZ for males was 38% lower than that of females, while WAZ was nearly 3 times lower (Table 4). Likewise, prevalence of stunting among males was more than double that of females. Other anthropometric measures—WLZ and BMIZ—show a similar pattern (higher values for females than males), although the differences were not statistically significant. Prevalences of underweight and wasting were low in this sample, and no statistically significant differences between groups were detected. Plasma concentrations of DMG for males were 24% lower than that of females. No significant differences were noted for other biomarkers, including choline, betaine, ratio betaine to choline, vitamin B12, LA, ALA, Ratio LA to ALA, and DHA.

**Table 4:** Nutrition status by sex.

Characteristics	Females (*n* = 77)	Males (*n* = 89)	*P* value^a^
**Anthropometry**			
Length-for-age *z*	− 1.56 ± 0.80	− 2.16 ± 1.07	0.000
Weight-for-age *z*	− 0.33 ± 0.93	− 0.86 ± 1.21	0.002
Weight-for-length *z*	0.83 ± 0.96	0.66 ± 1.06	0.264
Body mass index *z*	0.77 ± 0.99	0.55 ± 1.13	0.201
Stunted, n(%)	18 (23.4%)	45 (50.6%)	0.000
Underweight, n(%)	4 (5.2%)	10 (11.2%)	0.262
Wasted, n(%)	0 (0.0%)	1 (1.1%)	1.000
**Plasma concentration of biomarkers**			
Choline,^c^ µg/mL	2.93 ± 0.80	2.91 ± 0.80	0.870
Betaine,^c^ µg/mL	9.63 ± 3.87	8.88 ± 3.40	0.224
Ratio Betaine:Choline^c^	3.38 ± 1.27	3.20 ± 1.40	0.427
DMG,^c^ µg/mL	1.65 ± 1.20	1.25 ± 0.69	0.021
Vitamin B12,^b^ pmol/L	301.01 ± 143.18	320.14 ± 180.55	0.451
LA,^c^ µg/mL	1.19 ± 0.60	1.23 ± 0.74	0.717
ALA,^c^ µg/mL	16.34 ± 6.64	17.15 ± 9.55	0.558
Ratio LA:ALA^c^	14.84 ± 4.76	14.81 ± 3.56	0.961
DHA,^c^ µg/mL	1.58 ± 0.63	1.56 ± 0.85	0.904

Data are presented as mean ± standard deviation (SD) and Number (%).

^a^Two-sample *t* test for continuous variables and Fisher's exact test for categorical variables.

^b^*n* = 167.

^c^*n* = 142.

Based on our unadjusted model, LAZ for a male child is 0.63 units lower than for a female (95% confidence interval [CI] − 0.97, − 0.29; Table 5). After controlling for other variables in the model, LAZ for a male child is 0.62 units lower than for a female (95% CI − 0.98, − 0.26). For each unit increase in caregiver’s assessment of child’s appetite, LAZ increases by 0.38 (95% CI 0.05, 0.71). For each unit increase in vitamin B12, LAZ decreases by 0.002 (95% CI − 0.003, − 0.001). Other variables in the model showed no statistically significant relationship with LAZ. Added to our adjusted model, we found that an interaction between sex/gender and vitamin B12 was significant (*P* = 0.008). A graph of the joint effects with selected values for vitamin B12 is provided in Supplemental Fig. 1. For males, as vitamin B12 increases, LAZ decreases; however, for females, level of vitamin B12 has no association with LAZ. Prevalence ratios for unadjusted and adjusted models of the growth outcome stunting calculated using robust Poisson regression are presented in Supplemental Table 2.

**Table 5:** Linear regression analysis of infant feeding and biomarkers on growth outcome LAZ.

Predictor variable	Unadjusted			Adjusted		
	Coeff	95% CIs	*P* value	Coeff	95% CIs	*P* value
**Child**						
Male (reference = female)	− 0.63	− 0.97, − 0.29	0.000	− 0.62	− 0.98, − 0.26	0.001
Age, mo				− 0.09	− 0.25, 0.08	0.294
Firstborn				− 0.22	− 0.67, 0.22	0.321
**Maternal**						
Maternal age, y				0.00	− 0.03, 0.04	0.822
Education completed, y				0.04	− 0.02, 0.11	0.176
**Breastfeeding and complementary feeding**						
First 3 days fed something other than breastmilk				− 0.03	− 0.54, 0.49	0.916
Generally speaking, how is child's appetite when healthy?				0.38	0.05, 0.71	0.026
Dietary Diversity Score ≥ 4				0.16	− 0.21, 0.52	0.393
**Biomarkers**						
Choline, µg/mL				− 0.15	− 0.37, 0.07	0.184
Betaine, µg/mL				0.01	− 0.04, 0.06	0.699
DMG, µg/mL				0.03	− 0.16, 0.21	0.768
Vitamin B12, pmol/L				− 0.00	− 0.00, − 0.00	0.001
ALA, µg/mL				0.01	− 0.29, 0.30	0.963
DHA, µg/mL				− 0.09	− 0.36, 0.17	0.496
Intercept	− 1.54	− 1.79, − 1.29	0.000	− 1.18	− 3.35, 0.99	0.283
Adjusted *R*^2^	0.0832			0.1443		
*F (df, p-value)*	13.53 (1, 0.0003)			2.66 (14, 0.0020)		
*N*	139			139		

## Discussion

Among infants ages 6 to 9 months of age, males were at greater risk for stunting than females after adjusting for demographic, caregiver perceptions of appetite, and biological factors. Vitamin B12 showed a statistically significant negative association with LAZ. In terms of potential risk factors, this analysis shows that caregivers reported a healthy appetite among a higher proportion of boys compared to girls, but boys had lower odds of receiving certain complementary foods and dietary diversity compared to girls. These findings are consistent with the evidence that shows a biological health advantage for females; however, these results also suggest a gendered behavioral effect that favors girls. This is important to consider, as these results suggest a greater degree of vulnerability in male infants in this population.

Our findings of significantly worse growth outcomes for male infants align with the literature that reports a female advantage across a variety of health outcomes. Obstetrical complications, such as umbilical cord abnormalities, gestational diabetes, and delivery by cesarean section, are more common among male pregnancies than female^1,2,4^. Newborn males fare worse in susceptibility to birth trauma, intrauterine hypoxia and birth asphyxia, prematurity, respiratory distress syndrome, neonatal tetanus, congenital anomalies, intestinal infections, and lower respiratory infections^5^. Male children also appear to be more strongly affected by environmental exposures such as lead and pesticides, as well as by exposures resulting from maternal behaviors such alcohol or opioid use^3^. Although more research is needed to help elucidate the mechanisms underlying these outcomes, evolutionary theories of fitness suggest males face a trade-off between immunity and musculoskeletal maintenance, related to the major costs of immune response^23^. However, in a study examining the impact of immune function on growth among Amazonian forager-horticulturalists, investigators did not observe consistent differences between male and female children^24^. Others have explored evidence of sex-specific life history strategies, with females exhibiting greater growth canalization, as well as survivorship effects in the aftermath of unequal treatment in early life^25^.

When children receive equal treatment, female advantage is generally expected. When there are gendered differences in treatment, this trend may be either heightened, diminished, or even reversed depending on which gender is favored and to what degree. Thus, although female advantage may be the norm, it is highly context dependent. Alkema et al. (2014) identified 17 countries in 1990 with higher-than-expected under-5 mortality sex ratios and 5 countries in 2012: Guinea-Bissau, Kazakhstan, Mongolia, Uganda, and Uzbekistan. Similarly, a report by the United Nations Population Division (2011) identified twenty countries, including Ecuador, with excessively high infant mortality sex ratios (indicative of greater-than-expected male mortality)^5^. Excess female mortality has been reported in 10 countries based on outlying under-5 mortality sex ratios: Afghanistan, Bahrain, Bangladesh, China, Egypt, India, Iran, Jordan, Nepal, and Pakistan^6^. In India, the country with the highest excess female infant mortality rate in 2012^6^, nutritional disadvantage may play a role. An analysis of data from India’s National Family Health Survey (2005–2006) found that higher mortality risk among girls was associated with gendered differences in breastfeeding and complementary feeding; specifically, girls received less breast milk and less fresh milk than boys^8^.

We examined whether worse growth outcomes observed among male than female infants in Ecuador are related to gendered differences in breastfeeding or complementary feeding practices. Our results show a consistent pattern of gendered differences. Anthropological research conducted in Ecuador has reported that boys receive better treatment than girls during lactation and weaning, largely because of traditional beliefs that boys should be strong, while breastfeeding girls will convey undesirable male characteristics^10^. We found that boys were more often fed something other than breast milk within their first 3 days of life, as opposed to the recommended practice of exclusive breastfeeding for the first 6 months^26^. This could negatively impact infant health through increased immune challenge as the result of early exposure to contaminated liquids. Additionally, early supplementation negatively impacts breastmilk production, duration and exclusivity of breastfeeding, and the infant’s microbiome^27^. Boys were less likely to receive nutritious food items such as eggs, dairy products, and yellow fruits. However, boys were also less likely to receive water, thin porridge, or tea without milk, which are items of poor nutritional value. Boys were less likely to receive a score ≥ 4 for dietary diversity; this finding is significant because consumption of foods from 4 or more of the 7 constructed food groups is indicative of better quality diets regardless of breastfeeding status^18^.

Caretakers’ assessments of infants’ appetites were a statistically significant predictor of LAZ; perception of stronger appetite was associated with greater LAZ. Additionally, we noted gendered differences in caretakers’ perception of infants’ appetite, with poor appetite rarely noted for boys. Caretakers’ greater concern for poor appetite among girls may have led them to offer a wider variety of complementary foods, a potential explanation for the gendered differences observed here. Similarly, mothers in an indigenous village in Guatemala reported that infant boys were hungrier and less satisfied with breastfeeding alone, leading to earlier complementary feeding. While growth patterns initially favored female infants, sex differences declined in the second year of life, a pattern the authors attributed to gendered cultural perceptions around feeding^28^. In an Andean population of Peru, similar results were seen, with female infants initially showing better growth in height-for-age *z* scores at baseline, while differences by sex were not statistically significant at 6-month follow-up^29^. In Ecuador, additional research is needed to explore this result and track outcomes over time.

Among the biomarkers tested, only DMG showed a statistically significant difference by sex. Females had significantly higher DMG than males in this study. However, DMG was not a significant predictor of our growth outcome, LAZ. It is unclear whether DMG levels would be expected to differ by sex at this age. Vitamin B12 was identified as a significant predictor of LAZ. While seemingly counterintuitive, higher levels of vitamin B12 were associated with lower LAZ. However, for LAZ, the significant interaction between vitamin B12 and sex/gender indicated this association was specific to males but not females. To our knowledge, this is a novel finding, requiring additional research to verify and explain this result. Both DMG and vitamin B12 are components of the one-carbon metabolism cycle, impacting health and disease through their role in physiological processes including biosynthesis, amino acid homeostasis, epigenetic maintenance, and redox defense^30^.

## Limitations

This study was observational and not designed a priori to test sex and gender factors on nutrition outcomes in these infants. Several factors may have impacted growth outcomes but were not tested in this analysis. Birth weight and gestational age at birth were not collected because they may not be accurately remembered or may be unknown^11^. Intergenerational effects on linear growth have been well documented^31,32^, but we do not have maternal anthropometric measurements. We did not account for maternal nutritional status during pregnancy and breastfeeding nor recurrent infections in infants, which are reported to be proximal determinants of stunting^33^. We utilized baseline data to avoid the intervention effects of the RCT, but longitudinal data would be preferable; it is unclear if biomarkers and complementary feeding would be expected to predict growth outcomes at the same time point, rather than future growth. Additionally, we lack qualitative data which could have helped us understand the cultural beliefs underlying feeding practices.

## Conclusions

This analysis demonstrates that male infants in this Andean population fare significantly worse than females, both in terms of anthropometric growth outcomes and receipt of healthy complementary foods. That is, unlike earlier studies that showed that gendered differences in infant feeding favored boys, we found that those practices seem to favor girls, leaving boys at a double disadvantage. While further research is needed to confirm and explain this difference, this paper highlights the enhanced vulnerability of male infants in this study area. Moving forward, studies are needed to determine sex-specific ranges for biomarkers by age, and both biological mechanisms leading to different outcomes based on sex and differences based on social and cultural behaviors revolving around gender, which may impact nutritional outcomes, should be explored.

## Supplementary Information

Supplementary Table 1.

Supplementary Table 2.

Supplementary Figures.

Supplementary Legends.

## Data Availability

The data analyzed for this study are publicly available at Synapse, https://www.synapse.org/#!Synapse:syn21595086/files/.

## Abbreviations

ALA: Alpha-linolenic acid
BMIz: Body mass index *z* score
DHA: Docosahexaenoic acid
DMG: Dimethylglycine
LA: Linoleic acid
LAZ: Length-for-age *z* score
LC–MS/MS: Modified liquid chromatography-tandem mass spectrometry
RCT: Randomized Controlled Trial
SAGER: Sex and Gender Equity in Research
VIF: Variance inflation factor
WAZ: Weight-for-age *z* score
WLZ: Weight-for-length *z* score
