# Supplementary material for: A sex- and gender-based analysis of factors associated with linear growth in infants in Ecuadorian Andes
Source: Sci Rep. 2022 Feb 28;12:3292. doi: 10.1038/s41598-022-06806-3 (PMC8885924; doi:10.1038/s41598-022-06806-3)
Supplement: Supplementary file 1 — Supplementary Table 1. [file 41598_2022_6806_MOESM1_ESM.docx]

Supplemental Table 1. Health characteristics of study children by sex/gender^1^

| Health variable | Females  (*n* = 77) | Males  (*n*= 89) | *P* value^2^ |
| --- | --- | --- | --- |
| 6-month history of health service visits |  |  |  |
| Hospital | 22 (28.6%) | 23 (25.8%) | 0.733 |
| Health center, clinic | 46 (59.7%) | 57 (64.0%) |  |
| Private clinic | 2 (2.6%) | 5 (5.6%) |  |
| Other | 1 (1.3%) | 0 (0.0%) |  |
| Has not taken | 4 (5.2%) | 3 (3.4%) |  |
| Does not know | 2 (2.6%) | 1 (1.1%) |  |
| 6-month history of supplements or other nutrition products |  |  |  |
| Iron^3^ | 27 (35.5%) | 29 (33.0%) | 0.744 |
| Vitamin A^3^ | 22 (29.0%) | 36 (40.9%) | 0.141 |
| Multivitamin or Mineral^4^ | 19 (25.0%) | 19 (21.4%) | 0.584 |
| Food ration^5^ | 0 (0%) | 2 (2.33%) | 0.499 |
| 7-day symptom history |  |  |  |
| Fever^4^ | 28 (36.4%) | 29 (33.0%) | 0.743 |
| Diarrhea | 16 (20.8%) | 19 (21.4%) | 1.000 |
| Blood in stool | 0 (0.0%) | 2 (2.3%) | 0.500 |
| Skin rash | 20 (26.0%) | 33 (37.1%) | 0.136 |
| Constant cough^4^ | 30 (39.5%) | 46 (51.7%) | 0.121 |
| Congestion/runny nose | 36 (46.8%) | 41 (46.1%) | 1.000 |
| Panting/wheezing/difficulty breathing | 16 (20.8%) | 24 (27.0%) | 0.37 |
| Bruising, scrapes, or cuts^4^ | 17 (22.4%) | 13 (14.6%) | 0.228 |
| Toothache/teething | 6 (7.8%) | 12 (13.5%) | 0.319 |
| Given oral rehydration salts | 7 (9.1%) | 8 (9.0%) | 1.000 |

^1^Data are presented as number (%). ^2^Fisher's exact test for categorical variables. ^3^*n*=164. ^4^*n*=165. ^5^*n*=162.
