# Supplementary material for: A sex- and gender-based analysis of factors associated with linear growth in infants in Ecuadorian Andes
Source: Sci Rep. 2022 Feb 28;12:3292. doi: 10.1038/s41598-022-06806-3 (PMC8885924; doi:10.1038/s41598-022-06806-3)
Supplement: Supplementary file 2 — Supplementary Table 2. [file 41598_2022_6806_MOESM2_ESM.docx]

Supplemental Table 2. Prevalence ratios of stunting by selected factors^1^

|  | Unadjusted | | |  |  | Adjusted | | |
| --- | --- | --- | --- | --- | --- | --- | --- | --- |
| Predictor Variable | PR | 95% CIs | *P* value |  |  | PR | 95% CIs | *P* value |
| Child |  |  |  |  |  |  |  |  |
| Male (reference = female) | 2.37 | 1.42, 3.95 | 0.001 |  |  | 2.40 | 1.41, 4.08 | 0.001 |
| Age, mo | . | . | . |  |  | 1.13 | 0.94, 1.34 | 0.162 |
| Firstborn | . | . | . |  |  | 1.10 | 0.65, 1.87 | 0.709 |
| Maternal |  |  |  |  |  |  |  |  |
| Maternal age, y | . | . | . |  |  | 0.99 | 0.95, 1.03 | 0.642 |
| Education completed, y | . | . | . |  |  | 0.96 | 0.89, 1.02 | 0.201 |
| Breastfeeding and complementary feeding |  |  |  |  |  |  |  |  |
| First 3 days fed something other than breastmilk | . | . | . |  |  | 0.96 | 0.48, 1.90 | 0.905 |
| Generally speaking, how is child's appetite when healthy? | . | . | . |  |  | 0.75 | 0.47, 1.18 | 0.208 |
| Dietary Diversity Score ≥4 | . | . | . |  |  | 0.99 | 0.66, 1.49 | 0.977 |
| Biomarkers |  |  |  |  |  |  |  |  |
| Choline, µg/mL | . | . | . |  |  | 1.12 | 0.89, 1.44 | 0.368 |
| Betaine, µg/mL | . | . | . |  |  | 0.98 | 0.91, 1.06 | 0.677 |
| DMG, µg/mL | . | . | . |  |  | 0.81 | 0.61, 1.07 | 0.140 |
| Vitamin B12, pmol/L | . | . | . |  |  | 1.00 | 1.00, 1.00 | 0.011 |
| ALA, µg/mL | . | . | . |  |  | 0.87 | 0.60, 1.27 | 0.480 |
| DHA, µg/mL | . | . | . |  |  | 1.22 | 0.88, 1.69 | 0.230 |
| Intercept | 0.22 | 0.14, 0.35 | 0.000 |  |  | 0.19 | 0.01, 2.47 | 0.204 |
|  |  |  |  |  |  |  |  |  |
| *AIC* | 1.4781 | | |  |  | 1.6064 | | |
| *BIC* | -582.5605 | | |  |  | -526.5837 | | |
| *N* | 139 | | |  |  | 139 | | |

^1^Calculated with robust Poisson regression.
