# Supplementary figures and images for: A sex- and gender-based analysis of factors associated with linear growth in infants in Ecuadorian Andes
Source: Sci Rep. 2022 Feb 28;12:3292. doi: 10.1038/s41598-022-06806-3 (PMC8885924; doi:10.1038/s41598-022-06806-3)

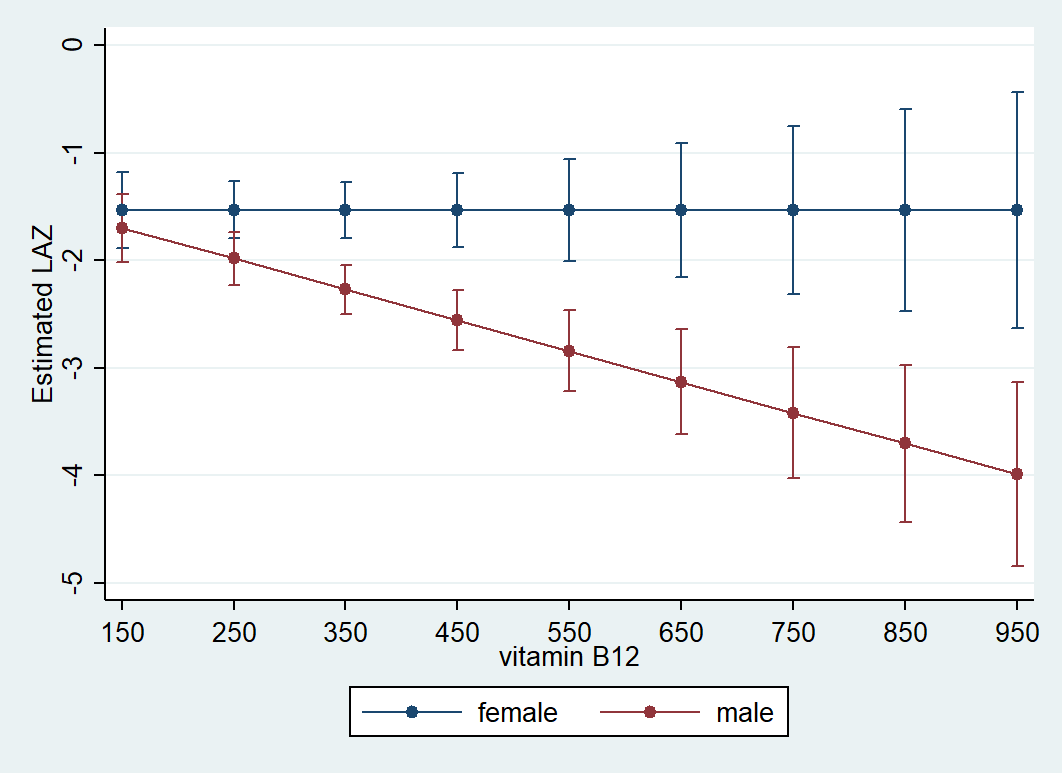

Supplement: Supplementary file 3 — Supplementary Figures. [file 41598_2022_6806_MOESM3_ESM.png]
