# Supplementary material for: A sex- and gender-based analysis of factors associated with linear growth in infants in Ecuadorian Andes
Source: Sci Rep. 2022 Feb 28;12:3292. doi: 10.1038/s41598-022-06806-3 (PMC8885924; doi:10.1038/s41598-022-06806-3)
Supplement: Supplementary file 4 — Supplementary Legends. [file 41598_2022_6806_MOESM4_ESM.docx]

**Figure Titles**

**Supplementary Figure 1**

Interactive effects of sex/gender and vitamin B12 on LAZ (length-for-age *z* score) with 95% CIs. This figure was created using Stata software version 14 (StataCorp. 2015. Stata Statistical Software: Release 14. College Station, TX: StataCorp LP.)
